# Electrostatic Gating of Monolayer Graphene by Concentrated Aqueous Electrolytes

**DOI:** 10.1021/acs.jpclett.3c00814

**Published:** 2023-05-01

**Authors:** Ghulam Abbas, Farjana J. Sonia, Martin Jindra, Jiří Červenka, Martin Kalbáč, Otakar Frank, Matěj Velický

**Affiliations:** † J. Heyrovský Institute of Physical Chemistry, Czech Academy of Sciences, Dolejškova 2155/3, 182 23 Prague, Czech Republic; ‡ FZU - Institute of Physics, Czech Academy of Sciences, Cukrovarnická 10/112, 162 00 Prague 6, Czech Republic; ¶ Department of Physical Chemistry, University of Chemistry and Technology, 16628 Prague, Czech Republic

## Abstract

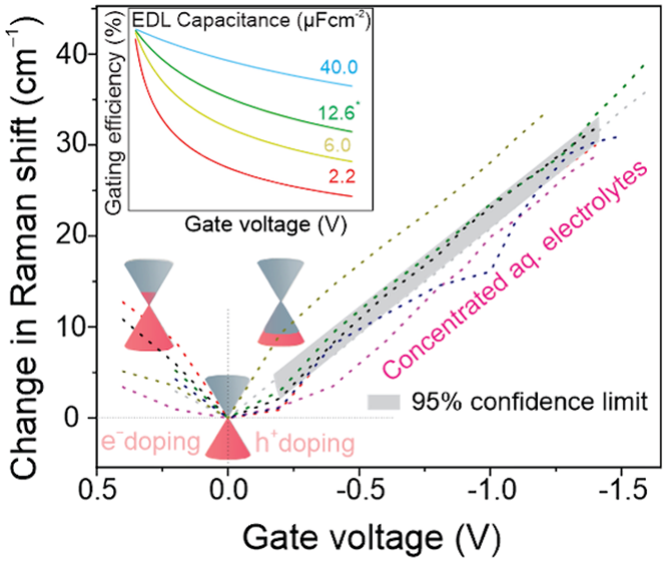

Electrostatic gating using electrolytes is a powerful approach for controlling the electronic properties of atomically thin two-dimensional materials such as graphene. However, the role of the ionic type, size, and concentration and the resulting gating efficiency is unclear due to the complex interplay of electrochemical processes and charge doping. Understanding these relationships facilitates the successful design of electrolyte gates and supercapacitors. To that end, we employ *in situ* Raman microspectroscopy combined with electrostatic gating using various concentrated aqueous electrolytes. We show that while the ionic type and concentration alter the initial doping state of graphene, they have no measurable influence over the rate of the doping of graphene with applied voltage in the high ionic strength limit of 3–15 M. Crucially, unlike for conventional dielectric gates, a large proportion of the applied voltage contributes to the Fermi level shift of graphene in concentrated electrolytes. We provide a practical overview of the doping efficiency for different gating systems.

The linear dispersion relationship between the energy and momentum of the charge carriers centered around the Dirac point makes graphene a zero-gap semiconductor with a linear dependence of the density of electronic states (DOS) on the Fermi level (*E*_F_). Since the first measurement of the electric field effect in graphene using a dielectric back gate,^1^ various electrostatic gating approaches have been used to study unique electronic properties of graphene.^2−4^ During nearly two decades of research, it has become apparent that gating using ionic electrolytes, including organic/aqueous solutions, ionic liquids, and solid polymer electrolytes, is among the most efficient ways to induce large densities of charge carriers in graphene.^5−7^ However, few systematic overviews exist of how various gate systems differ in their efficiency to induce electrostatic doping in graphene, particularly in the case of aqueous electrolytes.

Regardless of the type of gate employed, the mechanism of electrostatic doping remains the same: buildup of the charge upon application of a voltage between graphene and the gate electrode leads to a change in the charge carrier density in graphene and therefore a change in *E*_F_. The carrier density can be calculated from the applied voltage, quantum capacitance of graphene, and gate capacitance; however, this approach requires a knowledge of the thickness and permittivity of the gate medium.^1,8,9^ In contrast, Raman spectroscopy is a rapid and nondestructive method capable of probing the electronic structure and the charge carrier density in graphene. Consequently, Raman spectroscopy has been extensively applied to monitor the electrostatic doping of graphene.^4,8−11^ Changes in the Raman G mode and 2D mode frequencies, intensities, and widths correspond to the changes in the charge carrier density;^12,13^ however, it is necessary to discriminate these effects from lattice deformation, typically caused by strain.^14^

The explanation of the dependence of the Raman spectra on doping is not trivial and comprises the strong nonadiabatic coupling of phonons to electronic transitions across the gapless bands of graphene near the Dirac point, where |*E*_F_| equals half of the phonon energy (98 meV for the G mode), and adiabatic contributions of the changes in the lattice equilibrium parameter and coupling of phonons to higher energy transitions.^15,16^ For carrier densities corresponding to |*E*_F_| > 98 meV, the G mode stiffens almost symmetrically for both electron and hole doping. For carrier densities close to the Dirac point, the contribution of the phonon anomaly to the G mode frequency is mostly smeared out at room temperature, and only small changes can be observed.^8,9^ However, the strong electron–phonon coupling at small |*E*_F_| also causes phonons to decay into electron–hole pairs, giving rise to a shorter lifetimes and thus larger full widths at half-maximum (fwhm). For Fermi energies away from the phonon anomaly, the phonon decay is blocked according to the Pauli exclusion principle,^12,15,17^ and the G mode fwhm sharply decreases. For high doping levels, the intensity of the G mode sharply increases due to the partial blocking of the destructively interfering electronic transitions.^4,11,18^ The behavior of the 2D mode is also governed by the electron–phonon coupling and lattice expansion/contraction upon doping; however, the contribution of the former is smaller and the resulting 2D mode frequency shifts are markedly more asymmetric for electron/hole doping. The 2D mode is further influenced by the doping-dependent electron-electron scattering rate, which causes the intensity decrease and fwhm broadening upon carrier density increase.^19^ Furthermore, Raman spectroscopy also provides information about the structural defects, mechanical strain, and functionalization of graphene.^20^

The operating principle of electrostatic gating involves the creation of a capacitor between graphene and the gate electrode. The total charge built up in this capacitor, which determines the extent of electrostatic doping in graphene, is proportional to its capacitance. Furthermore, the low DOS of graphene gives rise to quantum capacitance,^21^ which uniquely affects the operation of the gate, as we will describe in detail later. Since the solid-state dielectrics have capacitances several orders of magnitude smaller (∼10^–9^–10^–8^ F cm^–2^) than those of the ionic electrolytes (∼10^–6^–10^–5^ F cm^–2^), substantially larger voltages are required to achieve the same carrier densities using the former (∼10–100 V) than the latter (∼0.1–1 V).^6^ However, it is difficult to accurately determine the gate capacitance, and therefore resulting doping effect, of an electrolyte gate due to uncertainty in the relative permittivity and Debye screening length. Moreover, the interpretation of the behavior of ions at the interface is a complex issue involving multiple effects. The type of electrolyte, ionic strength, and charge scattering have all been shown to affect the carrier density in graphene.^22−25^ Furthermore, concentrated electrolytes exhibit anomalous behavior, which leads to a breakdown of the classical decay relationship between the Debye screening length and ionic concentration.^26,27^ However, as we show here, the complexity of the interaction between the ionic species and graphene in terms of the resulting carrier density is in fact greatly reduced in highly concentrated aqueous electrolytes, when an external voltage is forced through the system.

In this work, we study the electrostatic gating of graphene and its efficiency using various aqueous electrolyte solutions of different concentrations. We investigate the shift rate in the Raman G mode of graphene, which is directly proportional to its Fermi level and therefore to the extent of doping, with the applied gate voltage. Our results show that the extent of charge doping of graphene is nearly identical for a range of highly concentrated electrolytes irrespective of their ionic strengths. Furthermore, we provide a general overview of the gating efficiency dependence on the gate capacitance and applied voltage in different gate media.

We perform *in situ* Raman spectroelectrochemistry of electrolyte-gated monolayer graphene in a microdroplet electrochemical cell, as shown schematically in Figure 1(a) for a range of aqueous electrolytes of different types and concentrations. The studied electrolytes include LiCl, LiClO_4_, Zn(ClO_4_)_2_, AlCl_3_, Al(ClO_4_)_3_, and Al(NO_3_)_3_. Monolayer graphene prepared by chemical vapor deposition was transferred onto a 300 nm SiO_2_/Si substrate and characterized by optical microscopy and Raman spectroscopy with 1.96 eV laser excitation (Supporting Figure S1). The main Raman modes of graphene were observed: G at ∼1587 cm^–1^ and 2D at ∼2653 cm^–1^. A microdroplet of electrolyte, 10–20 μm in diameter, using a potential in the range of −0.6 to 1.2 V vs Ag/AgCl, was formed with the help of a microcapillary (see Supporting Information for further details). One of the main advantages of the microdroplet measurements is the small current passed through the electrode, which reduces the undesirable contribution of uncompensated resistance to the applied voltage.

**Figure 1:**
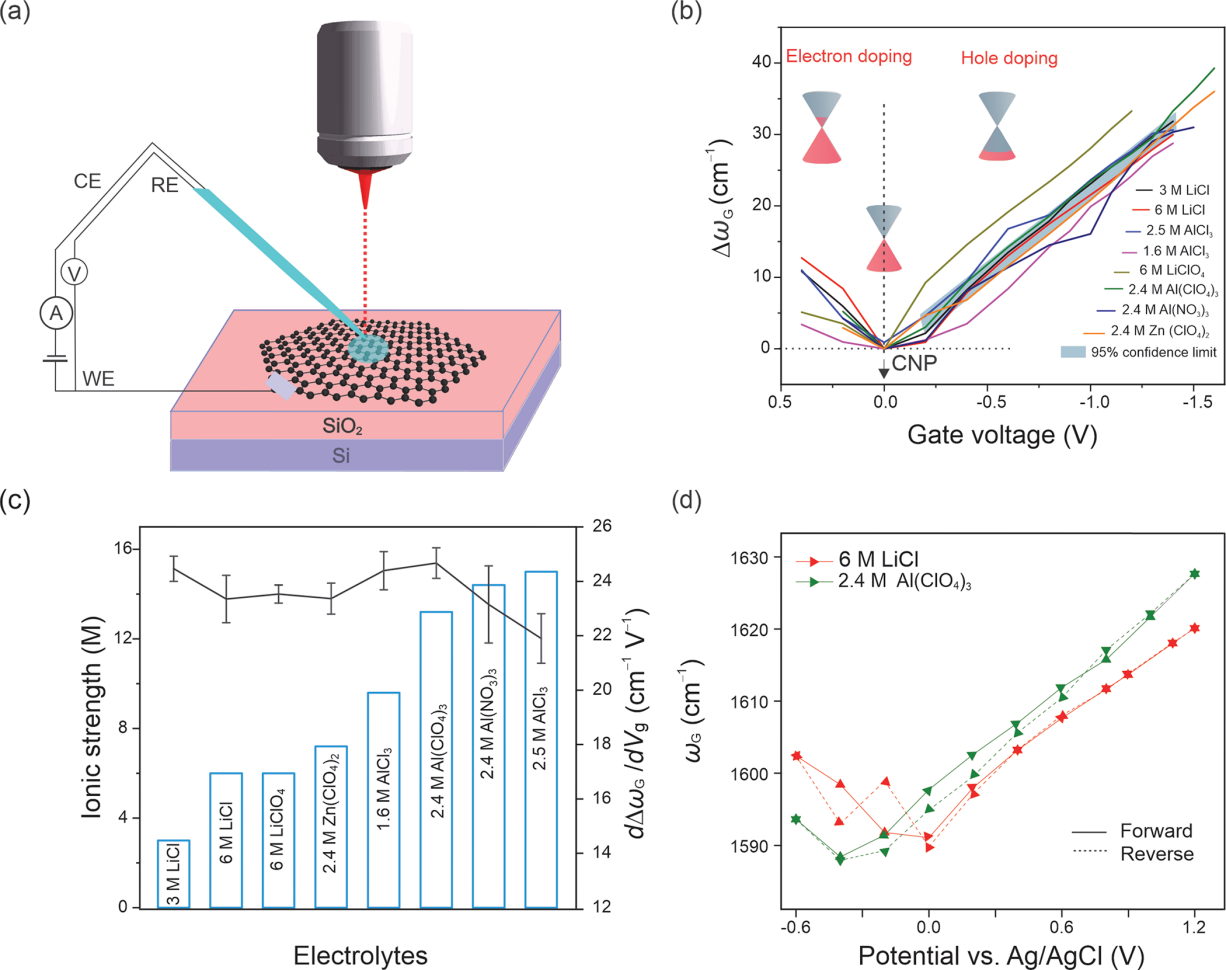
Electrostatic gating of graphene using different aqueous electrolytes. (a) Schematic of the *in situ* microdroplet Raman spectroelectrochemistry setup. (b) The shift of the G mode frequency for monolayer graphene, Δω_G_ = ω_G_ – ω_CNP_, as a function of the gate voltage, *V*_g_ = −(μ – μ_CNP_), recorded with a voltage step of 0.2 V. Δω_G_ corresponds to the charge doping of graphene relative to the CNP at the Dirac point. The confidence limit of 95% was calculated for *V*_g_ between −0.2 to −1.4 V. (c) The shift rate of the G mode with the gate voltage (*d*Δω_G_/*dV*_g_) compared for varying ionic strengths of different aqueous electrolytes. (d) Absolute frequency of the G mode for 6 M LiCl and 2.4 M Al(ClO_4_)_3_ as a function of the applied potential in one forward/reverse sweep cycle.

For each electrolyte type and concentration, we measured the spatial frequency of the Raman G mode (ω_G_, in cm^–1^) as a function of the applied electrode potential (μ). The electrostatic interactions between the electrolyte ions and graphene increase the density of electrons or holes in graphene, depending on the applied potential. The increased density of either of the carrier types leads to the stiffening of the G mode of graphene from its minimum doping value (ω_CNP_) at the charge neutrality point (CNP) where the carrier density is zero (equivalent to the Dirac point).^8^ We then subtract the CNP potential (μ_CNP_, where ω_G_ = ω_CNP_) from μ to define the gate voltage, *V*_g_ = −(μ – μ_CNP_), and plot the shift of the G mode frequency from its minimum at the CNP (Δω_G_ = ω_G_ – ω_CNP_) as a function of *V*_g_.

Figure 1(b) shows all the Δω_G_ dependencies on *V*_g_ measured in different electrolyte solutions, revealing the effects of the electrolyte and its ionic strength on electrostatic doping of graphene. By convention, negative gate voltages and positive electrochemical potentials correspond to hole doping, as shown in the inset of Figure 1(b). The average slope of the G mode shift dependency on potential (*d*Δω_G_/*dV*_g_) was calculated to be 23.0 ± 0.8 cm^–1^ V^–1^ considering all the hole doping data in Figure 1(b). The 95% confidence limit was calculated from the linear fit of G mode shift as a function of *V*_g_ from 0.2 to 1.4 V. The very narrow confidence limit implies that the ionic type and concentration have a negligible effect on the extent of the electrostatic gating of graphene in concentrated aqueous electrolytes.

This becomes even more apparent when comparing the *d*Δω_G_/*dV*_g_ values for different electrolyte solutions in Figure 1(c), which show little change with the ionic strength (*I* = 1/2∑_*i* = 1_^*n*^*c*_*i*_*z*_*i*_^2^, where *c*_*i*_ and *z*_*i*_ are the concentration and charge of the ion *i*, respectively). These striking observations suggest that, for a given *V*_g_, the electrolyte gating establishes electrical double layers with similar electrostatic doping efficiencies, irrespective of the type, concentration, and charge of the electrolyte ions. We also found the electrostatic gating using concentrated electrolytes to be reversible. Figure 1(d) shows ω_G_ for the forward (from low to high applied potentials) and reverse (from high to low applied potentials) gating using 6 M LiCl and 2.4 M Al(ClO_4_)_3_. The change in ω_G_ with potential exhibits only a negligible hysteresis during the hole doping.

While the effect of the gate voltage on doping is independent of the type and concentration of the electrolyte, the absolute position of the ω_G_ minimum on the applied potential axis clearly varies, as seen in Figure 1(d) and data for the full range of electrolytes shown in Supporting Figures S2 and S3. There are several likely origins of these shifts. First, the potential of the Ag/AgCl reference electrode used here depends on the chloride concentration, which is largely unknown in nonchloride electrolytes, and the applied potential will therefore vary by 59 mV for each 10-fold change in chloride concentration.^28^ Second, specific interactions of different types of ions at the graphene surface will determine the initial doping state before the application of potential as reported earlier.^23,24^ Third, charged impurities or trapped transfer residues change graphene’s surface potential and facilitate Coulombic scattering of carriers in graphene,^22,29,30^ which are, in turn, screened by the surrounding media, including electrolytes.^23,31,32^

Figure 2 details the evolution of the Raman G and 2D modes with applied gate voltage for a 2.4 M Al(ClO_4_)_3_ aqueous electrolyte. We already discussed the upshift of the G mode frequency with the hole doping, which is shown to increase by 39.3 cm^–1^ from 0.0 to 1.6 V in Figure 2(a). The intensities of both G and 2D modes also show a strong dependence on the *V*_g_. Specifically, the G mode intensity increases upon hole doping (Figure 2(a)), while the 2D mode intensity shows the opposite trend (Figure 2(b)), in accordance with the literature.^8,9,11,13^ It has previously been shown that the G mode intensity has a maximum at the Fermi level approximately equal to one-half of the average between the incoming photon energy (i.e., the laser excitation, *E*_ex_ = 1.959 eV) and the outgoing photon energy (*E*_ex_ – *ℏ*Ω_G_). The term *ℏ*Ω_G_, where *ℏ* is the reduced Planck constant and Ω_G_ is the angular frequency of the G mode, represents the energy of the scattered phonon.^11,18^ This G mode intensity maximum therefore corresponds to 2|*E*_F_| = *E*_ex_ – *ℏ*Ω_G_/2 = 1.861 eV and |*E*_F_| = 0.930 eV, where *ℏ*Ω_G_/2 = 98 meV for our minimum of Ω_G_ (equivalent to ω_G_ = 1587 cm^–1^). The increase of the G band intensity at 2|*E*_F_| = *E*_ex_ – *ℏ*Ω_G_/2 is caused by blocking electronic transitions due to filling (emptying) states in the conduction (valence) band, thereby canceling part of the destructively interfering Raman pathways.^11,18^ Although we do not reach a clear maximum in Figure 2(c) because we are limited by the oxidative branch of the electrochemical potential window, the G mode intensity indeed appears to plateau at the largest *V*_g_ of 1.6 V, which we therefore tentatively assign as corresponding to *E*_F_ of −0.930 eV. We also note that the exact value of the *E*_F_, at which the G band intensity reaches the maximum, will be slightly more negative than the value simply determined from 2|*E*_F_| = *E*_ex_ – *ℏ*Ω_G_/2 due to electron-hole asymmetry of the band structure in the *K*-*M* direction.^18^

**Figure 2:**
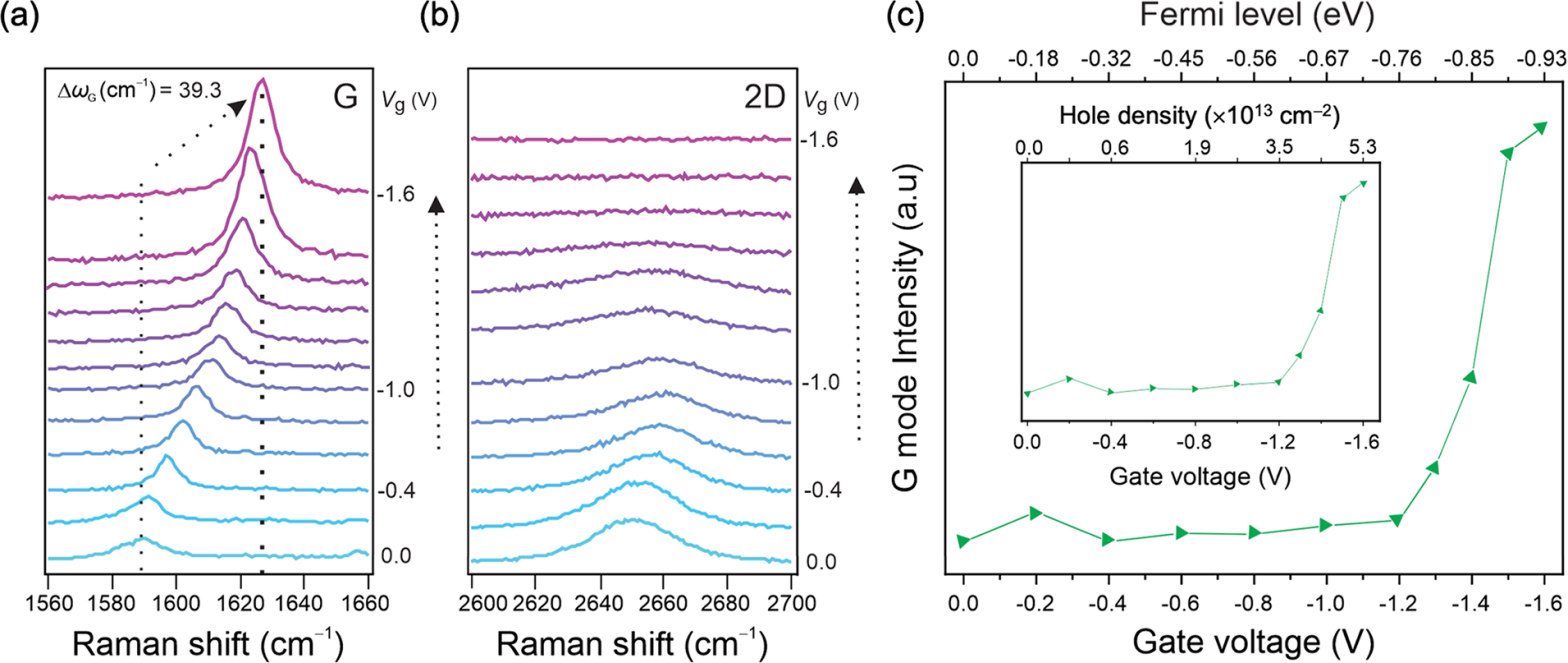
Evolution of the Raman spectra of monolayer graphene with applied gate voltage between 0.0 and 1.6 V using a 2.4 M Al(ClO_4_)_3_ aqueous electrolyte. (a) G mode of graphene as a function of *V*_g_. (b) 2D mode of graphene as a function of *V*_g_. (c) Change in the intensity of the G mode with the *V*_g_. The top horizontal axis shows the Fermi level relative to the Dirac point of graphene. The inset shows the same G mode intensity evolution but with the top axis recalculated as hole density using eq 2.

We also recorded other parameters as a function of gating, which are reported in the Supporting Information. The fwhm of the G mode decreases with an increase in both electron and hole density in graphene for small gate voltages, as shown in Supporting Figure S4, which is consistent with previous studies.^8,10,17^ The correlation between the 2D and G mode frequencies and the dependencies of the 2D mode fwhm and the 2D/G mode intensity ratio on potential are shown in Supporting Figures S4 and S5.

Let us now discuss the mechanism of the electrostatic doping by the electrolyte gate. Before applying the gate voltage, the ions are randomly distributed in the solution as shown schematically in Figure 3(a). The initial position of *E*_F_ with respect to the Dirac point  is affected by the defects/impurities in graphene and the adsorbed ions. Application of the gate voltage creates an electrostatic difference between graphene and the gate electrode (ϕ), which polarizes the graphene/electrolyte interface. This leads to charge accumulation and formation of an electrical double layer (EDL) of ions and counterions at the electrolyte side of the interface, as shown schematically in Figure 3(b). The emergence of EDL induces accumulation of the opposite-sign charge carriers on the graphene side of the interface, which in turn shifts the graphene Fermi level.^33^ The gating process can be described by the following relationship:^8,9,21^
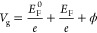
1where *e* is the elementary charge. *E*_F_ of graphene depends on its charge carrier density (*n*, positive for electrons and negative for holes) and Fermi velocity *v*_F_ as follows:^10,34^
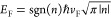
2while the potential difference ϕ can be expressed as the ratio of the total accumulated charge (*q* = *ne*) and gate capacitance (*C*_g_):^8^
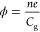
3

**Figure 3:**
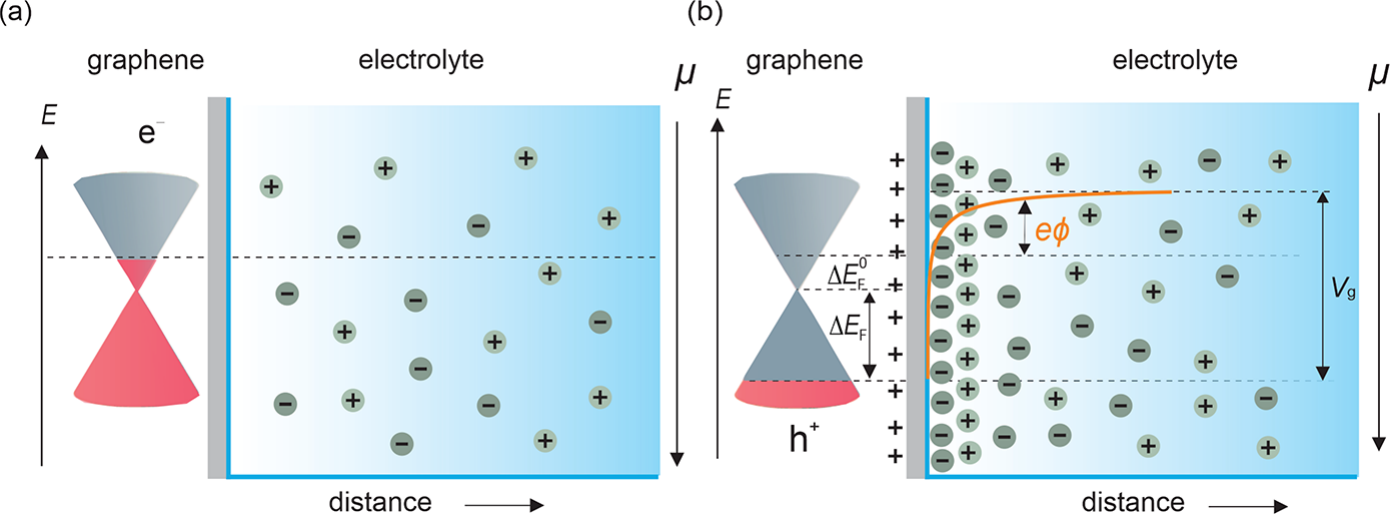
Schematic changes in the Fermi level of monolayer graphene electrostatically gated using an aqueous electrolyte. (a) Energy and potential levels in graphene and electrolyte in the absence of applied potential. (b) Energy and potential levels in graphene and electrolyte upon applying potential.

*C*_g_ is equivalent with the electrical double-layer capacitance (*C*_EDL_) for an electrolyte gate. The high concentration of ions in concentrated electrolytes results in efficient charge screening, which compresses the EDL to a typical width of ca. 1 nm corresponding to the Debye length (λ_D_).^5,6,27^ This creates an efficient gate capacitor capable of inducing sizable charge carrier densities in graphene with only moderate voltages, as discussed earlier. The resulting *C*_EDL_ of this capacitor, normalized per unit area, is inversely proportional to λ_D_ and can be approximated as
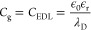
4where ϵ_0_ is the vacuum permittivity and ϵ_r_ is the relative permittivity of the electrolyte solution.

In order to understand the electrostatic doping of graphene, one also has to consider the quantum capacitance (*C*_Q_), arising from the low DOS and, subsequently, inefficient charge screening in graphene. *C*_Q_ is a special case of the space charge capacitance for a two-dimensional electron gas system.^21^ For *E*_F_ (relative to the Dirac point), which is substantially larger than the thermal energy *kT*, quantum capacitance can be approximated as follows:^35^
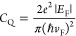
5

While most reports agree that *v*_F_ equals ∼1.1 × 10^6^ m s^–1^,^34^ larger values of up to ∼3 × 10^6^ m s^–1^ were reported depending on the media surrounding graphene.^36^Figure 4(a) shows the linear relationship between the quantum capacitance and Fermi level of graphene for the hole doping branch of the gating calculated from eq 5 (black) and reported by Zhan et al.^37^ (brown) who used a first-principles approach to determine the derivative of charge with respect to potential. The linear fit of the latter data (excluding a nonlinear region within 100 mV of the Dirac point) yields a slope nearly 2 times larger than that calculated from eq 5. This difference most likely originates from the varying estimation of the graphene DOS represented by 1/*v*_F_ in eq 5 and the one directly calculated from density functional theory in ref (37). Meanwhile, experiments reported by Xia et al.^29^ (purple) show a significant deviation from both calculations around the Dirac point but convergence toward the Zhan et al. data at the highest potentials.

**Figure 4:**
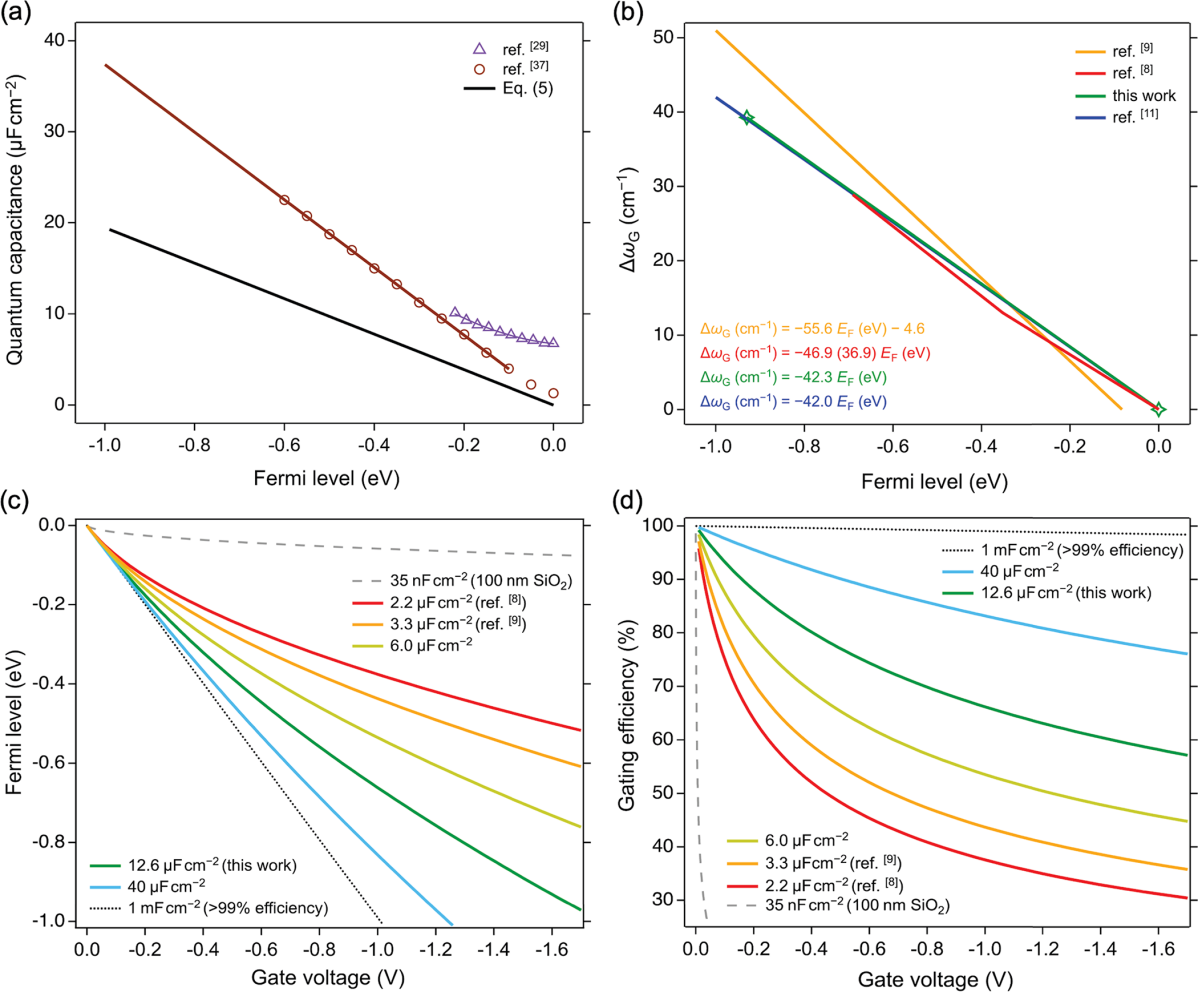
Quantum capacitance, Fermi level, and gating efficiency of electrolyte gating of graphene. (a) Dependence of graphene *C*_Q_ on *E*_F_. (b) Dependence of the shift of the Raman G mode frequency on *E*_F_. The green markers highlight the fact that the only two data points were used to estimate the Δω_G_ – *E*_F_ dependence from our results. (c) Dependence of graphene *E*_F_ on *V*_g_ for a range of different *C*_EDL_ values. (d) Dependence of the gating efficiency (|*E*_F_/*V*_g_| × 100%) on the *V*_g_ for a range of different *C*_EDL_ values. The Fermi levels are relative to the Dirac point in graphene.

From a practical perspective, it is particularly useful to evaluate the dependence of Δω_*G*_ on *E*_F_, which reflects the strength of the electron-phonon coupling in graphene^9^ and allows for a determination of the Fermi level of graphene using Raman spectroscopy. Figure 4(b) shows the Δω_*G*_ dependences on the Fermi level of graphene reported by Froehlicher and Berciaud (yellow),^9^ Das et al. (red),^8^ Chen et al. (blue),^11^ and our data (green) based on the estimate of *E*_F_ = 0.930 eV at the maximum Δω_G_ = 39.3 cm^–1^ (and *E*_F_ = 0.0 eV at Δω_G_ = 0.0 cm^–1^) for 2.4 M Al(ClO_4_)_3_ electrolyte. Note that Das et al.^8^ reported a Δω_G_ – *E*_F_ dependence with two linear segments of differing slopes. The discrepancies in the determination of the Δω_G_ dependence on *E*_F_ in Figure 4(b) are most likely rooted in the unreliable determination of the gate capacitance, which affects the gating efficiency and whose knowledge is necessary to accurately determine *E*_F_ from *V*_g_ according to eq 1. For example, Froehlicher and Berciaud^9^ employed a dual-gate setup and the known dielectric capacitance to determine *C*_EDL_, while Das et al.^8^ used eq 4 and literature values of ϵ_r_ and λ_D_ to accomplish the same. Importantly, all of these reports (including ours) neglect the dependence of *C*_EDL_ on *V*_g_, which will inevitably dictate the exact nature of these relationships. In reality, *C*_EDL_ is known to depend nonmonotonously on potential in different electrolytes and at different surfaces, with a several-fold difference between the lows and highs.^38^

A naïve way to understand eq 1 is that the energy released (spent) by applying *V*_g_ is split among compensating for the initial doping of graphene , raising (lowering) the Fermi level of graphene (*E*_F_), and charging of the interface (ϕ). A clear distinction between *E*_F_ and ϕ as depending on quantum capacitance and gate capacitance, respectively, was made previously.^8,9^ However, both of the terms, in fact, depend on the quantum capacitance of graphene, which is evident by combining eqs 1–3 and eq 5 (ignoring *E*_F_^0^ for simplicity):
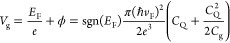
6

Nonetheless, due to the inverse dependence in the second term in eq 6, the larger the gate capacitance, the smaller the proportion of the gate voltage utilized to charge the interface and the larger the proportion of the gate voltage directed to shift the Fermi level. Thanks to the efficient charge screening and large *C*_EDL_ in a concentrated electrolyte, the system behaves as if the gate electrode was positioned within a Debye length of the graphene surface.

The quadratic dependence of the second term in eq 6 on *C*_Q_ fundamentally limits the ability to access high doping, even for the most efficient gating approaches. This is illustrated by our analysis using eq 6 shown in Figure 4(c), which reveals the dependence of *E*_F_ on *V*_g_ for several electrolytes with differing *C*_EDL_. The electrolyte gate with one of the lowest *C*_EDL_ (2.2 μF cm^–2^, red curve),^8^ quickly deviates from the ideal 1:1 linear proportionality between *E*_F_ and *V*_g_ (black dotted line), as *V*_g_ increases. The most optimistic case of an electrolyte with one of the highest *C*_EDL_ measured experimentally (40 μF cm^–2^, blue curve)^38^ follows the 1:1 line more closely but also deviates from it at high gate voltages. Our estimate of *E*_F_ from the G mode intensity maximum using eq 6 yields *C*_EDL_ = 12.6 μF cm^–2^, which is similar to commonly measured values in highly concentrated aqueous electrolytes^39^ and results in a *E*_F_-*V*_g_ response between the two previous extremes. In contrast to the electrolyte gates, the Fermi level of graphene for a typical 100 nm SiO_2_ dielectric gate with a gate capacitance of 35 nF cm^–2^ depends very weakly on the gate voltage (gray dashed curve).

Expressing the performance of the electrolyte gate as the gating efficiency dependence on gate voltage is even more instructive, as shown in Figure 4(d). This analysis shows that at *V*_g_ = 1.0 V, the gating efficiency drops below 70% for our work (12.6 μF cm^–2^) and below 40% for Das et al. (2.2 μF cm^–2^).^8^ For comparison, the gating efficiency of a 100 nm SiO_2_ dielectric layer (35 nF cm^–2^) drops as low as 6% at *V*_g_ = 1.0 V (17% at *V*_g_ = 0.1 V), contrasting the hypothetical 1 mF cm^–2^ capacitor with gating efficiency larger than 99% up to *V*_g_ = 1.0 V. Insights from Figure 4(c,d) explain why the high carrier density regime beyond ±1 × 10^14^ cm^–2^ (corresponding to |*E*_F_| > 1.3 eV) remains mostly elusive, despite the progress in electrolyte gating approaches made in the past decade.

Furthermore, the rise of the charge screening efficiency in highly concentrated electrolytes is severely limited by the ionic interaction arising from the steric effects such as charge overscreening, ion crowding, and ion pairing.^27,40^ Contrary to the prediction by classical models, the resulting reduction in charge screening paradoxically leads to an increase of the Debye length with increasing ionic concentration, which ultimately decreases the effective *C*_EDL_ according to eq 4. Such effects have been demonstrated both theoretically and experimentally for highly concentrated electrolytes.^26,41,42^ Importantly, the independence of the gating efficiency on the electrolyte type and concentration suggests that the effects caused by variations in *C*_EDL_ are smaller than the measurement errors.

In conclusion, electrostatic gating of monolayer graphene using highly concentrated aqueous electrolytes of varying ionic strength was interrogated using *in situ* Raman spectroelectrochemistry. The application of potential difference (gate voltage) through the electrolyte gate leads to electrostatic doping of graphene, which lowers or raises its Fermi level and manifests itself by changes in the position, shape, and intensity of the G and 2D Raman modes of graphene. Our results offer two critical insights into electrostatic gating using electrolytes. First, the rate of the G mode frequency change with the gate voltage (corrected for the Dirac point position) is the same, regardless of the electrolyte type, concentration, and ionic strength. This suggests that electrostatic doping is independent of the electrolyte properties, at least in the high ionic strength limit. Second, our analysis demonstrates that while electrolyte gating is the most effective approach to alter the Fermi level of graphene and induce high charge carrier density, it is not without limits. Even for electrolytes with highly efficient charge screening, the quantum capacitance fundamentally limits the maximum possible shift of the Fermi level with applied gate voltage. We demonstrate by comparison of different gating systems that this gating efficiency depends strongly on the gate capacitance and gate voltage, dropping as low as 70% for common electrolytes and applied voltages. Our findings have critical implications for electrolyte gating not only of graphene but also other materials with low DOS. Specifically, large ionic strengths can facilitate high charge doping regimes, through which exotic features of the electronic band structure, such as van Hove singularities, can be probed.^43^ Additionally, electrochemistry studies of low DOS materials, from which conclusions are drawn using the relative position of the applied potential and electronic band structure of the material, should be thoughtfully reconsidered.

## Data Availability

The data and analyses underlying this study are openly available in HeyRACK repository at https://doi.org/10.48700/datst.tgf5z-9fv07.

## Supporting Information Available

Experimental details, optical microscopy, and Raman spectroscopy of CVD graphene, G mode frequencies, G mode frequency shifts, and G and 2D mode fwhm’s as a function of the applied potential and gate voltage for all electrolytes, 2D–G mode vector analysis for all electrolytes, 2D to G mode intensity ratio as a function of the applied potential for 6 M LiCl and 2.4 M Al(ClO_4_)_3_ (PDF)
Transparent Peer Review report available (PDF)
