# Supplementary material for: Electrostatic Gating of Monolayer Graphene by Concentrated Aqueous Electrolytes
Source: J Phys Chem Lett. 2023 May 1;14(18):4281–8. doi: 10.1021/acs.jpclett.3c00814 (PMC10184166; doi:10.1021/acs.jpclett.3c00814)
Supplement: Supplementary file 1 — jz3c00814_si_001.pdf [file jz3c00814_si_001.pdf]

Supporting Information:

Electrostatic Gating of Monolayer Graphene by Concentrated Aqueous Electrolytes

Ghulam Abbas,[†,‡] Farjana j.Sonia,[†] Martin Jindra,[†,¶] Jiří Červenka,[‡] Martin Kalbáč,[†] Otakar Frank,[*,†] and Matěj Velický[*,†]

†*J. Heyrovský Institute of Physical Chemistry, Czech Academy of Sciences, Dolejškova 2155/3, 182 23 Prague, Czech Republic*

‡*FZU - Institute of Physics, Czech Academy of Sciences, Cukrovarnická 10/112, 162 00 Prague 6, Czech Republic*

¶*Department of Physical Chemistry, University of Chemistry and Technology, 16628 Prague, Czech Republic*

E-mail: otakar.frank@jh-inst.cas.cz; matej.velicky@jh-inst.cas.cz

Experimental

Monolayer graphene was prepared by chemical vapor deposition (CVD) on a Cu foil as a substrate. The polycrystalline Cu foil was annealed at 900 °C – 1000 °C under the continuous flow of H_2 for 20 minutes.[1] The graphene was grown under the flow of 1 standard cubic centimeter per minute (sccm) of CH_4 for 45 minutes and was then annealed for 5 minutes in H_2 atmosphere. Afterward it was cooled to room temperature.[2,3] The as-grown graphene was transferred to a cleaned Si/SiO_2 substrate of 300 nm thickness using the nitrocellulose polymer as reported elsewhere.[3] The transferred monolayer graphene was characterized by LabRAM HR spectrometer (Horiba Jobin-Yvon) using a 633 nm (1.96 eV) laser and 1 mW power at the sample with a diffraction grating of 600 l/mm and point-to-point spectral resolution was 1.8 cm^{-1}. An ultra-long working distance objective (Mitutoyo, NA 0.7) of 100x was operated via an Olympus BX47 confocal microscope. The recorded spectra were fitted with Lorentzian line shapes. The same setup was used for spectroelectrochemical measurements.

CVD graphene on Si/SiO_2 was connected to a Cu wire (Goodfellow, 99.9% purity) using an Ag conductive paste (Electrolube) and was used as a working electrode for the micro-droplet *in-situ* spectroelectrochemistry measurements. The microelectrochemical cell consists of a microcapillary with a tip of $\sim$ 1 µm diameter filled with 6 M LiCl aqueous electrolyte (LiCl, Sigma-Aldrich, 99.9%) and contains Ag/AgCl wire as reference electrode and Pt wire (Goodfellow, 99.99% purity) as the counter electrode.

The monovalent aqueous solutions of 3 M and 6 M LiCl, and 6 M $LiClO_4$ were prepared by dissolving LiCl and $LiClO_4$ salts (Sigma- Aldrich, 99.9%) in deionized water and bivalent 2.4 M $Zn(ClO_4)_2$ and 2.4 M $Zn(SO_4)$ aqueous solutions were prepared by dissolving $Zn(ClO_4)_2{\cdot}6$ H_2O (Alfa Aesar, Haverhill, MA, USA) and $Zn(SO_4).H_2O$ (Sigma- Aldrich, 98%) in deionized water. The multivalent 1.6 M and 2.5 M $AlCl_3$ and 2.4 M $Al(ClO_4)_3$ and 2.4 M $Al(NO_3)_3$ aqueous solutions were prepared by dissolving $AlCl_3{\cdot}6H_2O$ (Sigma- Aldrich, 99.0%) and $Al(ClO_4)_3{\cdot}9$ H_2O (Alfa Aesar, Haverhill, MA, USA) and $Al(NO_3)_3.9H_2O$ (Sigma- Aldrich,

98.0%) in deionized water. In addition, a micromanipulator and a picopump were used to control the shape, volume and diameter of the droplet with the flow of Ar gas.[4] The electrode potential (-0.6 V to 1.2 V) was applied with the potential step of 0.2 V using the Autolab PGSTAT30 (Metrohm).

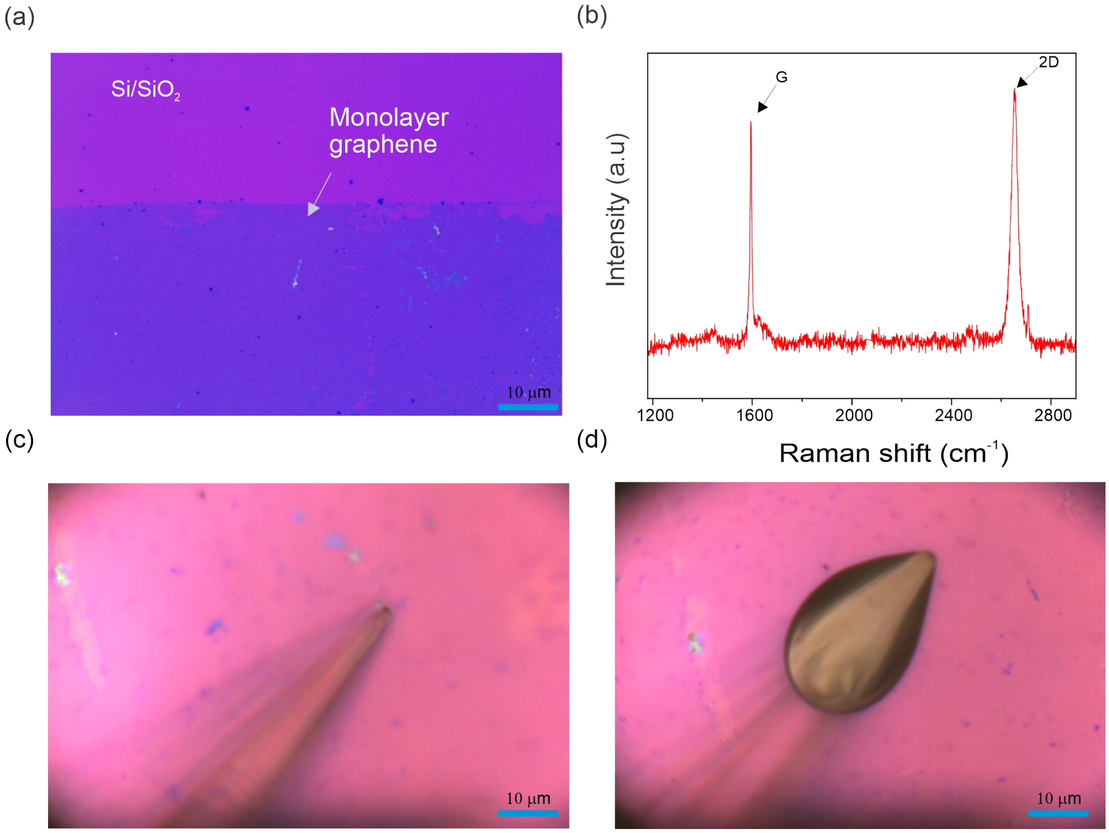

Figure S1: Characterization of CVD graphene. (a) Optical microscope image of monolayer graphene. (b) Raman spectrum of monolayer graphene. (c) Optical image of the microcapillary on graphene surface and (d) application of microdroplet.

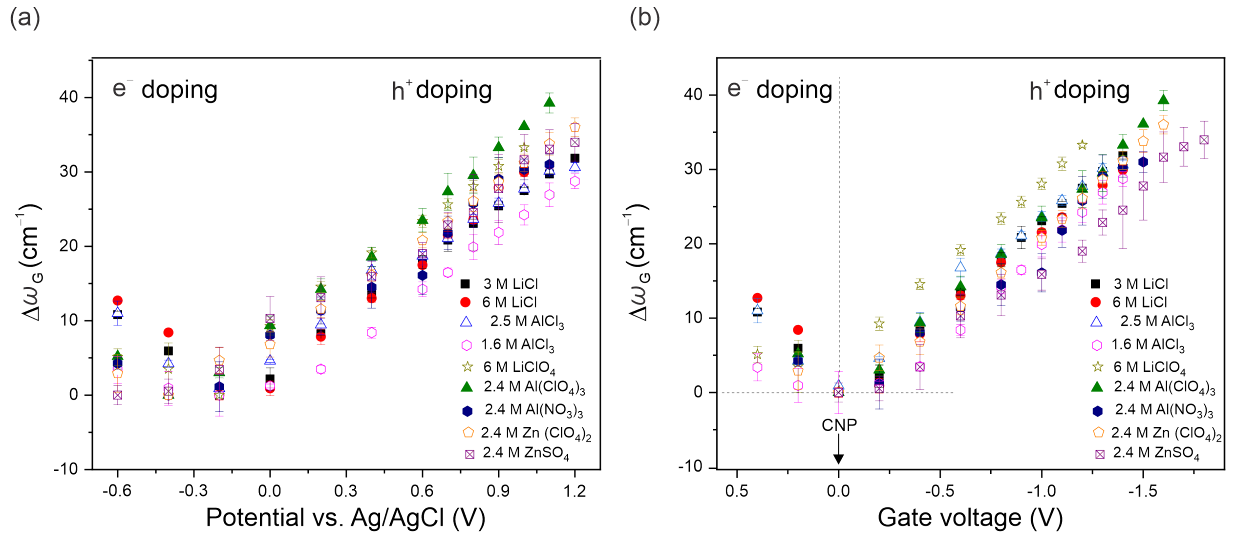

Figure S2: Electrochemical doping of monolayer graphene. (a) The shift in the G mode frequency ($\Delta\omega_{\mathrm{G}}$) of graphene in different electrolytes as a function of applied potential (-0.6 to 1.2 V). (b) The shift in the G mode frequency of monolayer graphene, $\Delta\omega_{\mathrm{G}} = \omega_{\mathrm{G}} - \omega_{\mathrm{CNP}}$, as a function of the applied gate voltage, $V_{\mathrm{G}} = -(\mu - \mu_{\mathrm{CNP}})$, recorded with voltage step of 0.2 V. $\Delta\omega_{\mathrm{G}}$ corresponds to charge doping of graphene relative to the CNP at the Dirac point.

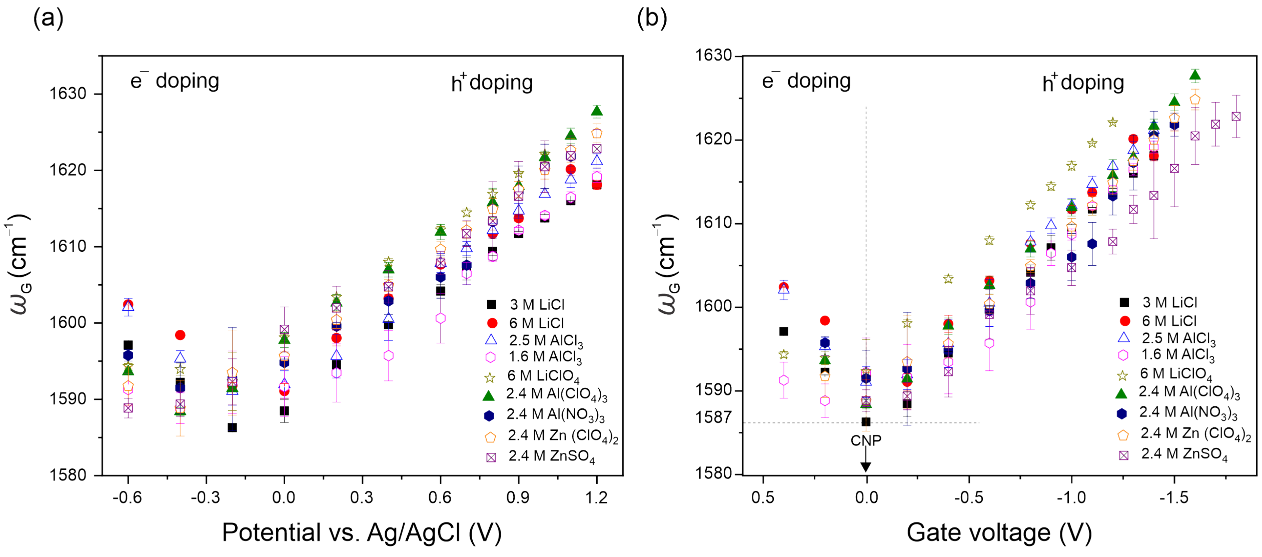

Figure S3: The G mode frequency of graphene ω_{G}) as a function of applied potential (-0.6 to 1.2 V) in different electrolytes solutions. (a) The G mode frequency as a function of the applied potential (-0.6 to 1.2 V). (b) The G mode frequency of graphene as a function of the applied gate voltage in different electrolyte solutions.

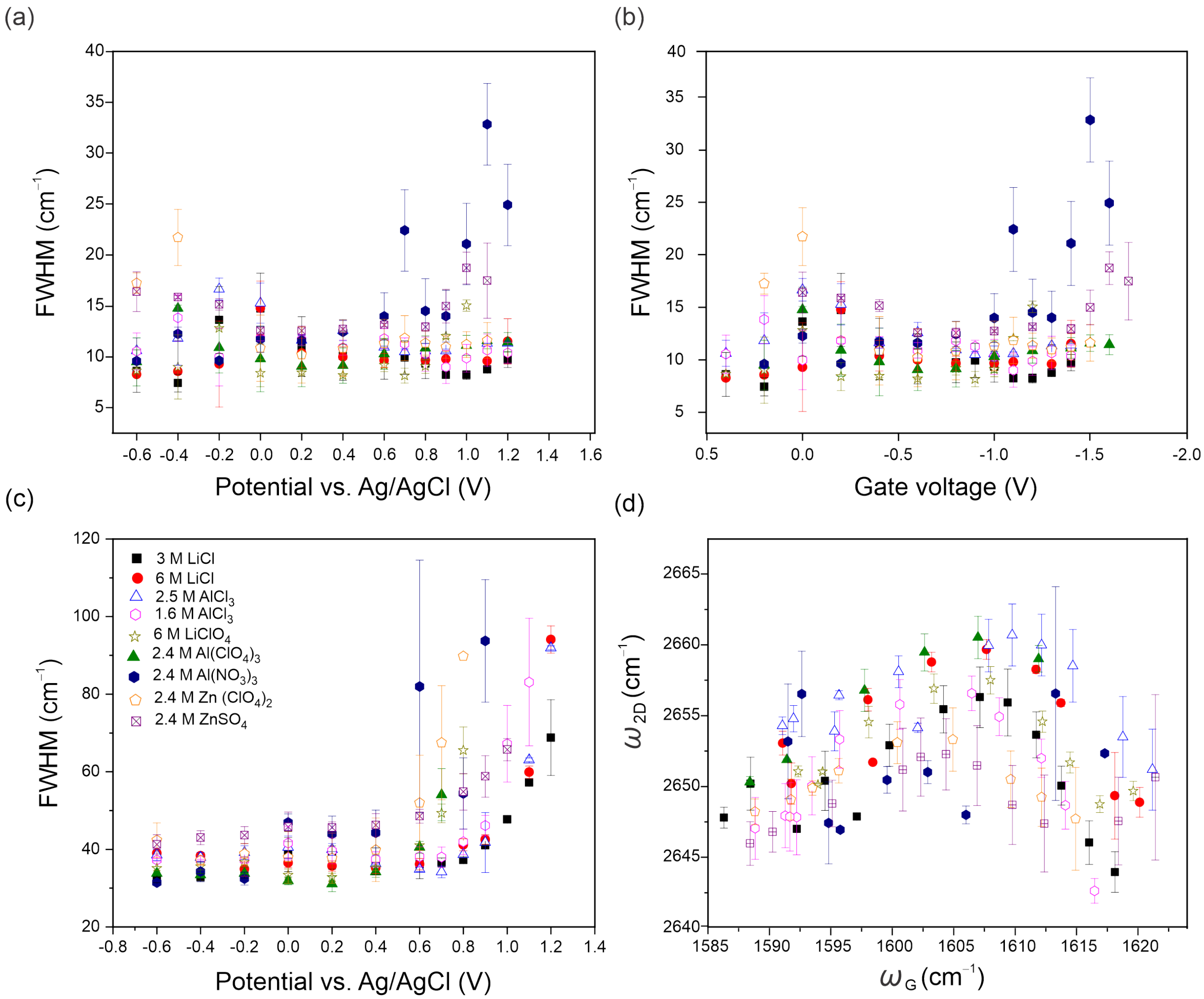

Figure S4: (a) FWHM of the G mode of graphene as a function of the applied potential (–0.6 to 1.2 V) for different electrolyte solutions. (b) FWHM of the G mode of graphene at CNP with respect to the applied gate voltage for different electrolyte solutions. (c) FWHM of the 2D mode of graphene as a function of applied potential (–0.6 to 1.2 V). (d) Correlation between the 2D mode frequency and the G mode frequency of graphene for different electrolyte solutions.

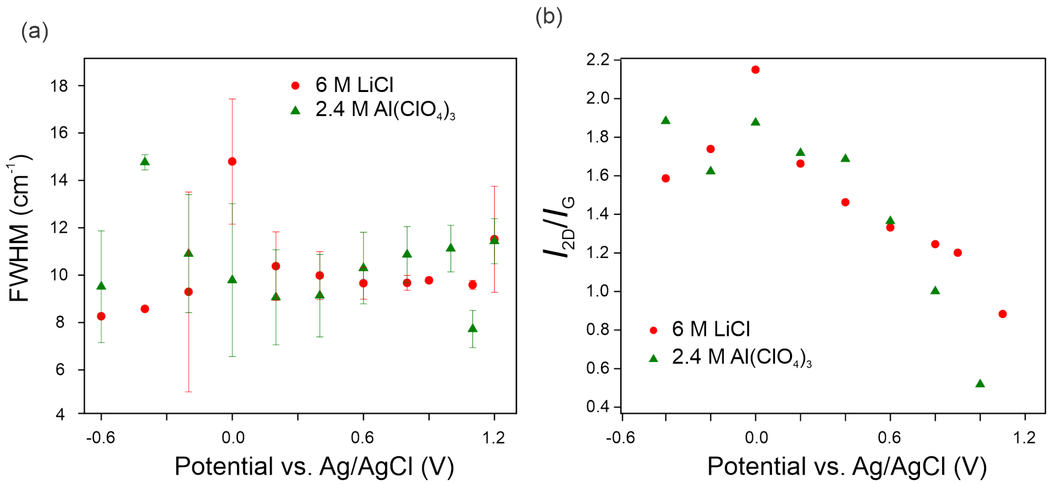

Figure S5: FWHM of the G mode of graphene as a function of potential using two different electrolyte solutions. (b) The change in the 2D to G mode intensity ratio (I_{2D}/I_G) with applied potential (–0.6 to 1.2V).

References

(1) Verhagen, T.; Pacakova, B.; Bousa, M.; Hübner, U.; Kalbac, M.; Vejpravova, J.; Frank, O. Superlattice in Collapsed Graphene wrinkles. *Sci. Rep.* **2019**, *9*, 1–7.

(2) Matulková, I.; Kovaříček, P.; Šlouf, M.; Němec, I.; Kalbáč, M. Surface Enhanced Infrared Absorption Spectroscopy for Graphene Functionalization on Copper. *Carbon* **2017**, *124*, 250–255.

(3) Hallam, T.; Berner, N. C.; Yim, C.; Duesberg, G. S. Strain, Bubbles, Dirt, and Folds: A Study of Graphene Polymer-Assisted Transfer. *Adv. Mater. Interfaces* **2014**, *1*, 1400115.

(4) Velicky, M.; Bradley, D. F.; Cooper, A. J.; Hill, E. W.; Kinloch, I. A.; Mishchenko, A.; Novoselov, K. S.; Patten, H. V.; Toth, P. S.; Valota, A. T., et al. Electron Transfer kinetics on Mono-and Multilayer Graphene. *ACS Nano* **2014**, *8*, 10089–10100.
