# Supplementary material for: Electrostatic Gating of Monolayer Graphene by Concentrated Aqueous Electrolytes
Source: J Phys Chem Lett. 2023 May 1;14(18):4281–8. doi: 10.1021/acs.jpclett.3c00814 (PMC10184166; doi:10.1021/acs.jpclett.3c00814)
Supplement: Supplementary file 2 — jz3c00814_si_002.pdf [file jz3c00814_si_002.pdf]

jz-2023-008144.R1

Name: Peer Review Information for "Electrostatic Gating of Monolayer Graphene by Concentrated Aqueous Electrolytes"

First Round of Reviewer Comments

Reviewer: 1

Comments to the Author

In the manuscript “Electrostatic gating of monolayer graphene by concentrated aqueous electrolytes” the authors explore the electrostatic gating of CVD monolayer graphene by means of Raman spectroscopy. By observing changes in the Raman G and 2D modes of the working graphene electrode upon applied potentials in a microdroplet system, authors determined that the type of the ion, its concentration, and charge have a negligible effect on the electrostatic gating of graphene by the concentrated electrolyte. The authors then model the graphene-electrolyte interface and compare their results with the literature data. In this section the authors highlight an inclusion of the impact of the applied potential on the capacitance of the electrical double layer in their model as compared to literature. The modelling supports experimental results. Combined together, this leads to the conclusion that the gating efficiency of the concentrated electrolyte does not depend on its properties, as the applied potential has the major contribution. These results are important for development of sensor systems and supercapacitors, as well as fundamental studies of materials with low density of states.

The text is written in a clear and concise language. The manuscript has sufficient number of illustrations to demonstrate the experimental details and results, modelling results, and schematics of the system. Experimental procedures and supplementary data are thoroughly described in the Supporting Information.

Good quality of the data and its presentation makes this manuscript fit for publication in the ACS Journal of Physical Chemistry Letters.

Reviewer: 2

Comments to the Author

The paper by G.Abbas et al. presents an investigation of the effect of electrolyte gating on the Raman spectra when working in electrolytes of high concentration (> 1.5 M) or electrolytes with high ionic strength (> 3 M). This was possible in a microdroplet generated using a micro capillary with integrated counter and reference electrodes. The entire graphene sheet was connected as the working electrode. The observed changes in the position and FWHM of the Raman modes are consistent with several previous reports of electrolyte gated Raman measurements.

In this paper manly two conclusions were drawn. At the high ionic strength (3 M - 15 M) used, the rate of shift of the Raman G-band was found to be rather constant. Secondly, based on a detailed analysis of the balance between the electrical double layer (EDL) capacitance and the quantum capacitance, it is concluded that the major proportion of the applied gate voltage directly modulates the position of the Fermi level.

While the results are interesting as such, they are not surprising or new. It is well-known that the EDL and quantum contributions occur in series at the graphene-electrolyte interface. At a very high ionic strength the EDL thickness becomes quite small resulting in a large EDL capactiance. Hence naively one would expect that the gate voltage modulation can indeed shift the Fermi level efficiently. Also it is a little surprise that the rate of shift is similar between the used electrolytes, since several of the cations / anions are known not to specifically interact with graphene in the potential range used. Nevertheless, the study is well carried out, and the data are well presented.

I have just a few suggestions for improvement.

*) In the abstract, it sounds as if it is very generic that the rate of change in doping with gate voltage is totally independent of ionic strength (due to the use of the strong phrase 'no measurable influence'). It is important to stress already in the abstract that this conclusion applies especially to high ionic strength and the investigated range of ionic strength needs to be mentioned in the abstract.

*) In conclusion it is mentioned that the observations have strong implications for electrolyte gating - Here it is not so clear for what applications we need such a high ionic strength. It would be necessary to suggest some examples here and explain a bit more what implication is implied here.

Author's Response to Peer Review Comments:

Dear Prof. Editor,

I confirm that we have fully addressed both the requested non-scientific editorial changes as well as the issues raised by reviewer 2, namely:

*) In the abstract, it sounds as if it is very generic that the rate of change in doping with gate voltage is totally independent of ionic strength (due to the use of the strong phrase 'no measurable influence'). It is important to stress already in the abstract that this conclusion applies especially to high ionic strength and the investigated range of ionic strength needs to be mentioned in the abstract.

We have specified the ionic strength range in the abstract and conclusions.

*) In conclusion it is mentioned that the observations have strong implications for electrolyte gating - Here it is not so clear for what applications we need such a high ionic strength. It would be necessary to suggest some examples here and explain a bit more what implication is implied here.

We have added an explanatory sentence addressing the potential implications of high ionic strength, which reads: Specifically, large ionic strengths can facilitate high charge doping regimes, through which exotic features of the electronic band structure, such as van Hove singularities, can be probed.

The changes made in the manuscript are highlighted in a separate file.

We hope the changes raised our manuscript to a sufficient standard for the J Phys Chem Lett.

Best regards,

Matej Velicky
